# The Role of ZAP and TRIM25 RNA Binding in Restricting Viral Translation

**DOI:** 10.3389/fcimb.2022.886929

**Published:** 2022-06-21

**Authors:** Emily Yang, LeAnn P. Nguyen, Carlyn A. Wisherop, Ryan L. Kan, Melody M.H. Li

**Affiliations:** ^1^ Molecular Biology Institute, University of California, Los Angeles, Los Angeles, CA, United States; ^2^ Department of Microbiology, Immunology and Molecular Genetics, University of California, Los Angeles, Los Angeles, CA, United States; ^3^ Department of Biological Chemistry, David Geffen School of Medicine, University of California, Los Angeles, Los Angeles, CA, United States; ^4^ AIDS Institute, David Geffen School of Medicine, University of California, Los Angeles, CA, United States

**Keywords:** ZAP, TRIM25, RNA binding, CpG sensing, translation inhibition, co-factors, alphavirus, Japanese encephalitis virus

## Abstract

The innate immune response controls the acute phase of virus infections; critical to this response is the induction of type I interferon (IFN) and resultant IFN-stimulated genes to establish an antiviral environment. One such gene, zinc finger antiviral protein (ZAP), is a potent antiviral factor that inhibits replication of diverse RNA and DNA viruses by binding preferentially to CpG-rich viral RNA. ZAP restricts alphaviruses and the flavivirus Japanese encephalitis virus (JEV) by inhibiting translation of their positive-sense RNA genomes. While ZAP residues important for RNA binding and CpG specificity have been identified by recent structural studies, their role in viral translation inhibition has yet to be characterized. Additionally, the ubiquitin E3 ligase tripartite motif-containing protein 25 (TRIM25) has recently been uncovered as a critical co-factor for ZAP’s suppression of alphavirus translation. While TRIM25 RNA binding is required for efficient TRIM25 ligase activity, its importance in the context of ZAP translation inhibition remains unclear. Here, we characterized the effects of ZAP and TRIM25 RNA binding on translation inhibition in the context of the prototype alphavirus Sindbis virus (SINV) and JEV. To do so, we generated a series of ZAP and TRIM25 RNA binding mutants, characterized loss of their binding to SINV genomic RNA, and assessed their ability to interact with each other and to suppress SINV replication, SINV translation, and JEV translation. We found that mutations compromising general RNA binding of ZAP and TRIM25 impact their ability to restrict SINV replication, but mutations specifically targeting ZAP CpG-mediated RNA binding have a greater effect on SINV and JEV translation inhibition. Interestingly, ZAP-TRIM25 interaction is a critical determinant of JEV translation inhibition. Taken together, these findings illuminate the contribution of RNA binding and co-factor interaction to the synergistic inhibition of viral translation by ZAP and TRIM25.

## Introduction

The type I interferon (IFN) response is one of the first lines of cellular defense against invading pathogens. The IFN-induced zinc finger antiviral protein (ZAP) is a potent inhibitor of diverse RNA and DNA viruses (Yang and Li, 2020; Ficarelli et al., 2021). ZAP encodes at least four splice isoforms, two of which, ZAPS (short) and ZAPL (long) are well characterized (Charron et al., 2013; Li et al., 2019; Schwerk et al., 2019). ZAPS and ZAPL share the N-terminal CCCH zinc fingers (ZnFs) that mediate RNA binding while ZAPL has an additional C-terminal catalytically inactive poly(ADP-ribose) polymerase (PARP)-like domain, which contributes to its greater antiviral activity compared to the IFN-inducible ZAPS (Yang and Li, 2020; Ficarelli et al., 2021). Two primary mechanisms of ZAP antiviral activity include targeting viral RNA for degradation and suppressing viral translation (Yang and Li, 2020). However, it remains largely unclear how ZAP is able to coordinate multiple means of viral antagonism while lacking enzymatic activity on its own. These mechanisms appear to be dependent on viral context. For example, ZAP is thought to inhibit human immunodeficiency virus-1 (HIV-1) primarily by targeting its RNA for degradation (Zhu et al., 2011). On the other hand, ZAP inhibits alphavirus replication by suppressing translation of incoming viral genomes and inhibits replication of Japanese encephalitis virus (JEV) by both RNA degradation and translation suppression (Bick et al., 2003; Chiu et al., 2018; Zhu et al., 2012). In addition to inhibiting viral replication by binding directly to viral RNA, ZAP also recruits cellular co-factors, such as exosome components, the putative endoribonuclease KHNYN, and the E3 ligase tripartite motif containing protein 25 (TRIM25) (Guo et al., 2007; Li et al., 2017; Zheng et al., 2017; Ficarelli et al., 2019).

Earlier efforts to elucidate ZAP RNA binding activity showed that ZAP binds RNA with its four N-terminal CCCH ZnFs, and mutations of ZnFs 2 and 4 most dramatically reduce ZAP antiviral activity (Guo et al., 2004). The first structural study of only the N-terminal region of ZAP (NZAP) posited that the four ZnFs form two distinct RNA binding cavities; however, this study did not directly show ZAP bound to RNA (Chen et al., 2012). In recent years, much focus has been given to the discovery of ZAP as a CpG dinucleotide sensor in the context of HIV-1 infection (Takata et al., 2017; Ficarelli et al., 2019). Since then, two studies have elucidated the structure of ZAP complexed with CpG-containing RNA and identified critical residues responsible for its CpG binding specificity (Meagher et al., 2019; Luo et al., 2020). While several studies have characterized ZAP RNA binding activity, they have done so in the context of only NZAP or only ZAPL and not ZAPS, and primarily focused on the mechanism of RNA degradation (Chen et al., 2012; Meagher et al., 2019; Luo et al., 2020; Gonçalves-Carneiro et al., 2021). Moreover, most did not utilize full-length viral RNA for measuring ZAP RNA binding activity, instead assaying with only a ZAP-sensitive fragment (Chen et al., 2012; Luo et al., 2020). Meanwhile, it has been suggested that translation inhibition may be preceded by and required for ZAP-mediated mRNA degradation (Zhu et al., 2012). Furthermore, these ZAP RNA binding studies have mostly expressed RNA binding mutants against the background of endogenous ZAP and TRIM25.

While much attention has been given to characterizing ZAP RNA binding, less has been given to its critical co-factors such as TRIM25. TRIM25 was identified as a ZAP co-factor in the context of inhibiting alphavirus translation (Li et al., 2017; Zheng et al., 2017). However, it remains unclear whether TRIM25 modulates the RNA binding activity or specificity of ZAP. Like ZAP, TRIM25 is also an RNA binding protein (Choudhury et al., 2017; Sanchez et al., 2018; Garcia-Moreno et al., 2019). TRIM25 RNA binding has been mapped to two separate motifs: a 39-amino acid stretch in the C-terminal PRY-SPRY domain (Choudhury et al., 2017), and a lysine-rich sequence (7K) within the L2 linker connecting the coiled-coil and PRY-SPRY domains (Sanchez et al., 2018). TRIM25 appears to preferentially bind G- and C-rich sequences, and prefers mRNAs and long intergenic non-coding RNAs (Choudhury et al., 2017). TRIM25 RNA binding is required for its ubiquitin ligase activity (Choudhury et al., 2017), which in turn is required for its function in ZAP antiviral activity (Li et al., 2017; Zheng et al., 2017). Both TRIM25 and ZAP directly bind SINV RNA, and have been demonstrated to associate more strongly with SINV RNA during infection (Guo et al., 2004; Garcia-Moreno et al., 2019). TRIM25 and ZAP also associate with one another, with the ZAP interaction motif within TRIM25 mapped to its C-terminal PRY-SPRY domain (Li et al., 2017). Meanwhile, the TRIM25 interaction motif for ZAP is thought to reside within its N-terminal ZnFs (Gonçalves-Carneiro et al., 2021), though the additional PARP-like domain within ZAPL may contribute to TRIM25 binding as well by modulating proper localization (Kmiec et al., 2021).

While some have attempted to illuminate the contribution of ZAP or TRIM25 RNA binding to the ZAP-TRIM25 interaction, these studies have focused on only a few select mutations. Still, most agree that RNA binding in either ZAP or TRIM25 is not required for the ZAP-TRIM25 interaction. One group demonstrated not only that RNase A treatment has little effect on the ZAPL-TRIM25 interaction, but also that an example ZAPL RNA binding mutant retains and even increases its interaction with TRIM25 (Gonçalves-Carneiro et al., 2021). Another showed the TRIM25 mutant in which the 7K motif is replaced with alanines (abbreviated as 7KA) associates more strongly with ZAP (Gonçalves-Carneiro et al., 2021), while a third found that the TRIM25 mutant with a deletion of the 39-amino acid sequence in the PRY-SPRY domain (abbreviated as ΔRBD) fails to bind ZAP at all (Choudhury et al., 2017). Still, it is important to note that the RNA binding motif deleted in TRIM25 ΔRBD is located within the same PRY-SPRY domain that TRIM25 uses to interact with ZAP, complicating these findings, and that the same study corroborated previous findings that RNase A treatment has little impact on the ZAP-TRIM25 interaction (Choudhury et al., 2017).

In light of the recent novel structural insights, we asked how different ZAP and TRIM25 RNA binding mutations affect the ability of ZAP and TRIM25 to interact with one another and to restrict SINV and JEV translation. We curated a panel of ZAP and TRIM25 RNA binding mutants from prior studies, including mutants with a range of RNA binding and antiviral capabilities (Guo et al., 2004; Chen et al., 2012; Meagher et al., 2019; Luo et al., 2020). We first characterized these ZAP and TRIM25 mutants’ direct binding to SINV RNA. We then asked how their ability to bind RNA affects their ability to interact with one another. Generally, we observed that ZAP mutants that fail to bind SINV RNA interact more strongly with TRIM25. In contrast, we observed that the KHNYN TRIM25 7KA mutant binds both SINV RNA and ZAP more strongly than TRIM25 wild-type (WT), and that the TRIM25 ΔRBD mutant is too unstable to have any detectable interaction with ZAP. Moreover, we generally found that mutants that fail to bind SINV RNA also fail to inhibit SINV replication and translation, with residues important for CpG recognition playing a critical role in ZAP translation inhibition. Surprisingly, when we tested the ability of ZAP and TRIM25 RNA binding mutants to inhibit translation of a JEV replicon, some mutants demonstrate increased antiviral activity, while those with mutations in residues important for CpG recognition have reduced activity. We then performed a correlation analysis to determine which ZAP properties are necessary for its antiviral activity against SINV and JEV. We found a significant negative correlation between ZAP SINV RNA binding and ZAP-TRIM25 interaction. We also found a significant positive correlation between ZAP SINV RNA binding and SINV replication inhibition, as well as between ZAP-TRIM25 interaction and JEV translation inhibition. These data together suggest that ZAP RNA binding and interaction with TRIM25 may form two distinct determinants for ZAP antiviral mechanisms in different viral contexts, even while they appear to be inversely correlated in the context of binding to SINV RNA. Altogether, this study furthers our understanding of how viral RNA binding and interaction with co-factors may modulate translation inhibition by ZAP.

## Results

### ZAP and TRIM25 RNA Binding Mutants Show a Range of Binding to SINV Genomic RNA

To investigate the role of ZAP and TRIM25 RNA binding in the context of viral translation, we generated a panel of constructs with mutations previously demonstrated to impact RNA binding. For ZAP, each of the following mutations was introduced in both ZAPS and ZAPL to probe potential isoform differences in RNA binding and antiviral function. These mutations fall into two general categories: 1) ZnF mutants that individually disrupt each CCCH motif and 2) CpG RNA binding cavity mutants. We made four individual mutations to disrupt each N-terminal CCCH ZnF: H86K, C88R, C168R, and H191R, which are located in ZnF 1, 2, 3, and 4, respectively (Guo et al., 2004). Two putative RNA binding cavities were previously identified based on a crystallized NZAP structure without bound RNA (Chen et al., 2012). Based on this study, we also made two triple mutations in the two predicted RNA binding cavities contained within the ZnFs: V72A/Y108A/F144A (abbreviated as VYF), found within ZnFs 2-3, and H176A/F184A/R189A (abbreviated as HFR), found within ZnF 4 (Chen et al., 2012). More recent studies have elucidated structures of NZAP bound to CpG-containing RNA (Meagher et al., 2019; Luo et al., 2020). Building on this work, we made two double mutations and one triple mutation within the ZnFs that mediate CpG dinucleotide-specific binding. These mutations are C96A/Y98A (abbreviated as CY) and K107A/Y108A (abbreviated as KY) within ZnF 2, and E148A/K151A/R170A (abbreviated as EKR) within ZnF 3, which demonstrate a range of RNA binding and antiviral activities (Meagher et al., 2019; Luo et al., 2020). NZAP mutants CY, KY, and EKR previously demonstrated loss of antiviral activity against a SINV NanoLuc luciferase reporter virus, with EKR exhibiting the least defect as compared to NZAP WT (Luo et al., 2020). Notably, the individual mutations Y108A/F and F144A/Y appear to be critical for ZAP recognition of CpG-rich RNA, wherein they completely or partially abolish ZAP’s ability to discriminate between CpG-rich and CpG-deficient strains of HIV-1, respectively (Meagher et al., 2019; Luo et al., 2020).

For TRIM25, we generated two constructs, one in which we replaced the lysine-rich motif in the L2 linker with alanines (TRIM25 7KA) and one in which we deleted the previously identified RNA binding domain in the PRY/SPRY domain (TRIM25 ΔRBD) (Choudhury et al., 2017; Sanchez et al., 2018).

**Figure 1:**
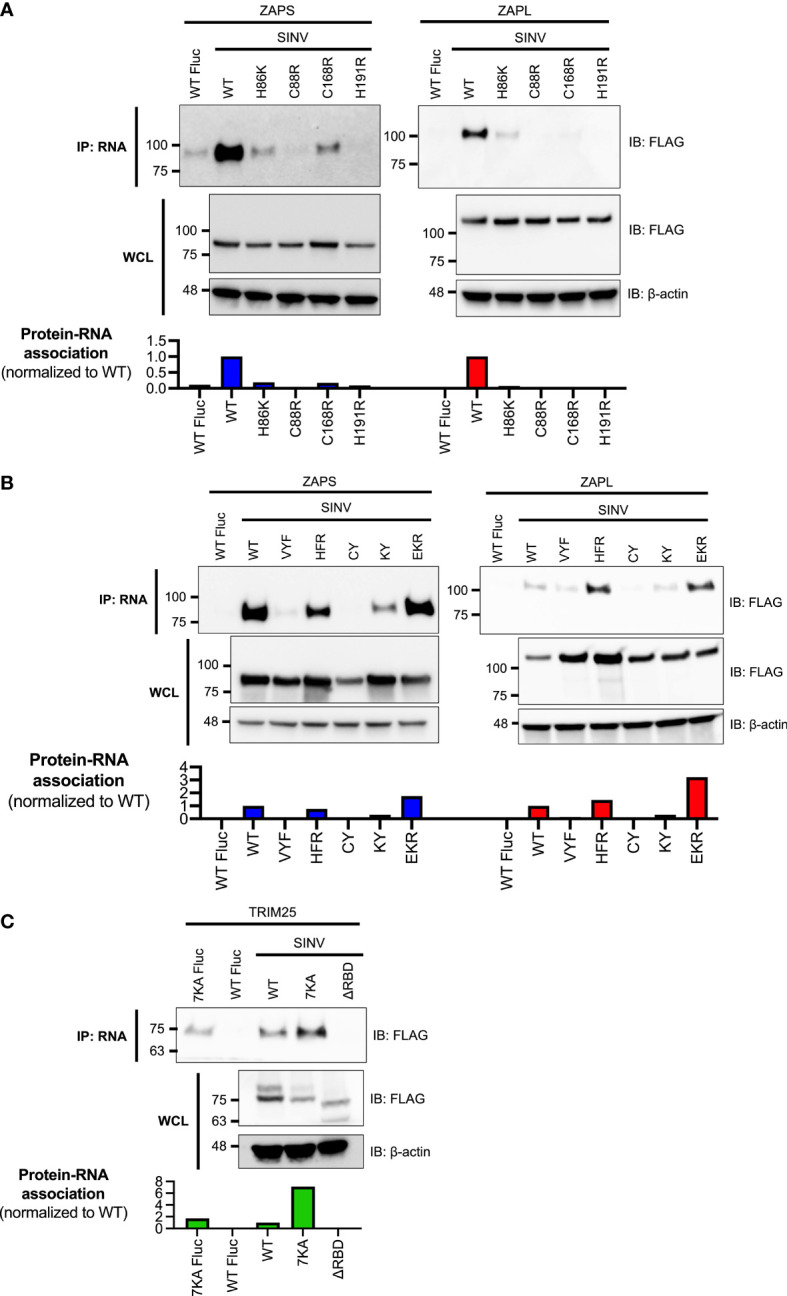
Association of ZAP and TRIM25 RNA binding mutants with SINV RNA. **(A, B)** ZAP KO or **(C)** TRIM25 KO 293T cells were transfected with **(A)** ZAP zinc finger (ZnF) mutants, **(B)** ZAP CpG RNA binding cavity mutants, or **(C)** TRIM25 mutants. ZAP or TRIM25 pulled down (IP) with Sindbis virus (SINV) or firefly luciferase (Fluc) RNA and in whole cell lysate (WCL) were assayed by immunoblot (IB). Blots were quantified with ImageJ. Data are representative of two independent experiments.

We cloned each of these ZAP and TRIM25 WT or RNA binding mutants into a pcDNA3.1-3XFLAG or -myc plasmid, allowing for transient expression of each construct following transfection into ZAP or TRIM25 KO 293T cells. We titrated the amount of plasmid to transfect for each construct to ensure even expression across constructs for ZAPS, ZAPL, and TRIM25 WT (**Supplemental Figure 1**). Because the ZAP CpG RNA binding cavity mutants generally express at higher levels than the ZnF mutants (**Supplemental Figure 1**), we decided to transfect two amounts of ZAPS and ZAPL WT in our assays to match these two expression patterns. We then assessed the ability of the ZAP and TRIM25 mutants to bind SINV genomic RNA by an *in vitro* RNA pull-down assay. We incubated lysates of cells transfected with ZAP or TRIM25 mutants with biotin-labeled SINV (Toto1101 strain) genomic RNA, allowing for RNA and bound protein to be immunoprecipitated using streptavidin beads and probed for the presence of bound ZAP or TRIM25. As a negative control, we also assessed the ability of the WT constructs to bind firefly luciferase (Fluc) RNA. We quantified the resultant ZAP and TRIM25 bound to RNA and normalized to input ZAP and TRIM25 protein levels with ImageJ (Davarinejad, 2015). The ZnF mutations in both ZAPS and ZAPL drastically reduce all SINV RNA binding, with only the ZAPS and ZAPL ZnF 1 mutant (H86K) and the ZAPS ZnF 3 mutant (C168R) showing low levels of binding (**Figure 1A**). In contrast, mutations in the CpG RNA binding cavities result in a range of binding phenotypes. For both ZAPS and ZAPL, the VYF, CY, and KY mutants show complete to near complete loss of SINV RNA binding, while the HFR and EKR mutants show similar or increased RNA binding relative to ZAPS and ZAPL WT (**Figure 1B**).

The TRIM25 mutants demonstrate diverging RNA binding phenotypes. The 7KA mutant not only has increased binding to SINV RNA relative to WT, but also to the Fluc negative control (**Figure 1C**). Consistent with previous findings, the ΔRBD mutation abolishes binding to SINV RNA (**Figure 1C**). Together, our findings indicate that the different RNA binding residues of ZAP and TRIM25 contribute in varying degrees to viral RNA binding.

### RNA Binding Mutations Generally Increase ZAP-TRIM25 Association

Given that TRIM25 RNA binding has been purported to stimulate its interaction with ZAP (Choudhury et al., 2017), and that the ZAP ZnFs responsible for RNA binding are also thought to mediate its interaction with TRIM25 (Gonçalves-Carneiro et al., 2021), we hypothesized that abolishing ZAP RNA binding would negatively impact ZAP association with TRIM25, and vice versa. We also aimed to capture any ZAP isoform-specific characteristics for association with TRIM25, given previous reports that TRIM25 preferentially interacts with ZAPL (Li et al., 2017; Kmiec et al., 2021). Therefore, we expected that ZAP RNA binding mutants would display decreased enrichment in the presence of TRIM25 co-immunoprecipitation (co-IP) compared to ZAP WT. In accordance with prior work and our observation of different RNA binding activities of the TRIM25 mutants (**Figure 1C**), we also expected that TRIM25 7KA and ΔRBD would behave differently.

**Figure 2:**
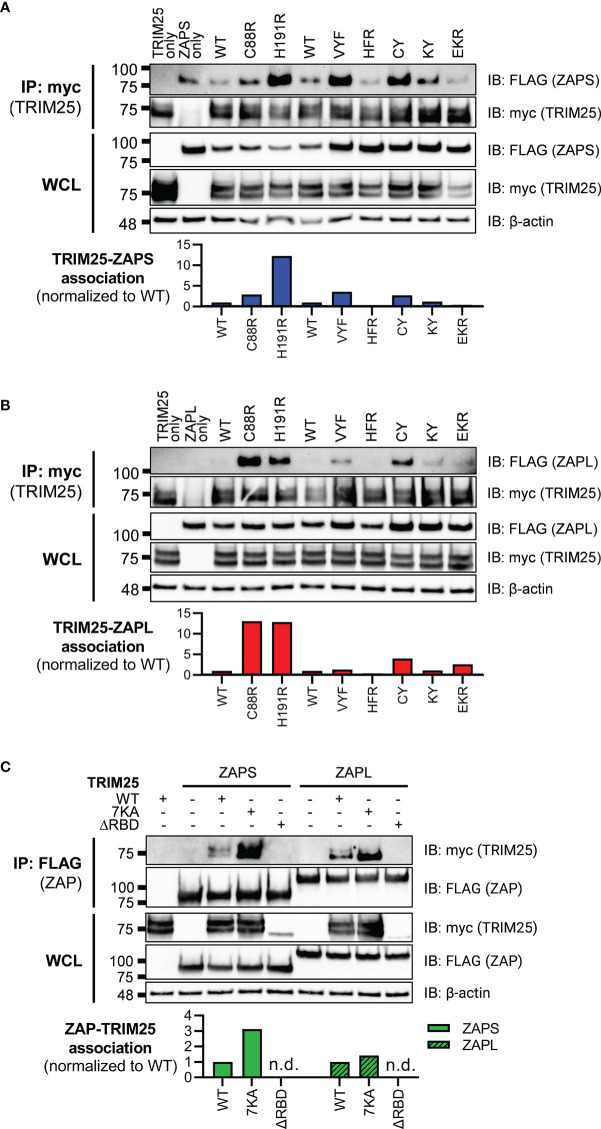
Interaction of ZAP or TRIM25 RNA binding mutants with TRIM25 or ZAP WT. **(A, B)** Western blot of ZAP KO 293T cells transfected with myc-tagged TRIM25 and **(A)** FLAG-tagged ZAPS RNA binding mutants or **(B)** FLAG-tagged ZAPL RNA binding mutants. Different amounts of **(A)** ZAPS and **(B)** ZAPL WT were transfected to match protein expression levels for each subset of ZnF mutants and CpG RNA binding cavity mutants. Blots were quantified with ImageJ. Data are representative of two independent experiments. **(C)** Western blot of TRIM25 KO 293T cells transfected with FLAG-tagged ZAPS or ZAPL and myc-tagged TRIM25 RNA binding mutants; n.d. stands for not detectable by western blot. Blots were quantified with ImageJ. Data are representative of two independent experiments.

To test this hypothesis, we transfected ZAP KO 293T cells with either FLAG-tagged ZAPS or ZAPL RNA binding mutants and myc-tagged TRIM25 WT. Given that the ZAP ZnFs 2-4 display approximately equal, near complete loss of RNA binding (**Figure 1A**), and that ZnFs 2 and 4 are more important for mediating ZAP antiviral activity against alphaviruses and for CpG specificity (Bick et al., 2003; Meagher et al., 2019; Luo et al., 2020), we proceeded with testing only ZnF mutants 2 and 4 in addition to the complete panel of CpG RNA binding cavity mutants. We then performed a myc IP to enrich for TRIM25 and probed for the presence of associated ZAPS or ZAPL, quantifying resultant ZAP pulldown with ImageJ (Davarinejad, 2015). Surprisingly, we found that both ZnF mutants (C88R and H191R) in both ZAPS and ZAPL display increased association with TRIM25 (**Figures 2A, B**). Of the remaining RNA binding mutants, VYF, CY, and KY with near complete loss of RNA binding (**Figure 1B**) display similar to increased TRIM25 association for both ZAPS and ZAPL, though to a lesser extent than the ZnF mutants (**Figures 2A, B**). Only HFR with unaffected or mildly reduced RNA binding (**Figure 1B**) exhibits diminished interaction with TRIM25 as compared to ZAPS and ZAPL WT (**Figures 2A, B**), while EKR which mostly retains or even gains binding to SINV RNA (**Figure 1B**) binds to TRIM25 to a similar degree as compared to ZAP WT (**Figures 2A, B**).

To test the ability of TRIM25 RNA binding mutants to interact with ZAP, we transfected TRIM25 KO 293T cells with myc-tagged TRIM25 RNA binding mutants and FLAG-tagged ZAPS or ZAPL WT. When we transfected amounts that would yield similar levels of TRIM25 expression (**Figure 1C**), we found that these levels are too low to visualize previously demonstrated interactions between TRIM25 WT and ZAPS or ZAPL (data not shown). In our hands, the TRIM25 ΔRBD mutant is markedly less stable than the other TRIM25 constructs. Therefore, we maximized the transfected amounts of all TRIM25 forms to better visualize any differences in TRIM25 association with ZAP, normalizing the TRIM25 co-IP to input lysates and quantifying with ImageJ (Davarinejad, 2015). As expected, we observed that TRIM25 7KA binds more robustly than TRIM25 WT to both ZAPS and ZAPL, while the TRIM25 ΔRBD levels are likely too low to be detected in the ZAP co-IP (**Figure 2C**).

Together, these data suggest that ZAP RNA binding may compete with ZAP-TRIM25 interaction likely because ZAP interacts with TRIM25 through its N-terminal ZnFs. Moreover, there do not appear to be ZAP isoform-specific differences for the RNA binding mutants as a whole, with trends of increased or decreased TRIM25 association holding true for each mutant in ZAPS and ZAPL.

### RNA Binding Is Required for Both ZAP and TRIM25 Inhibition of SINV Replication

**Figure 3:**
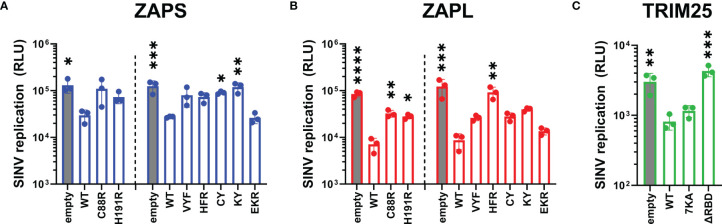
Inhibition of SINV replication by ZAP and TRIM25 RNA binding mutants. **(A, B)** ZAP KO 293T cells or **(C)** TRIM25 KO 293T cells were transfected with **(A)** ZAPS RNA binding mutants, **(B)** ZAPL RNA binding mutants, or **(C)** TRIM25 RNA binding mutants, infected with SINV Toto1101/luc at an MOI of 0.01 PFU/cell, and lysed 24 hours post infection (h.p.i.) for measurement of luciferase activity. Different amounts of **(A)** ZAPS and **(B)** ZAPL WT were transfected to match protein expression levels for each subset of ZnF mutants and CpG RNA binding cavity mutants. Data from triplicate wells are representative of two independent experiments. Asterisks indicate statistically significant differences as compared to **(A, B)** ZAP WT or **(C)** TRIM25 WT within each subset of RNA binding mutants (by one-way ANOVA and Dunnett’s multiple comparisons test: *p < 0.05; **p < 0.01; ***p < 0.001; ****p < 0.0001). Unlabeled comparisons are not significant.

Next, we asked how loss of RNA binding would affect the ability of ZAP and TRIM25 to inhibit SINV replication. Given the centrality of RNA binding to ZAP antiviral activity and to TRIM25 ligase activity, we hypothesized that mutations with near complete loss of RNA binding would abolish antiviral activity completely, while mutations with moderate loss of RNA binding would exhibit an intermediate phenotype. We transfected ZAP KO 293T cells with ZAP RNA binding mutants and infected with the SINV luciferase reporter virus Toto1101/luc. As expected, the mutant EKR which retains its ability to bind SINV RNA as compared to ZAPS and ZAPL WT (**Figure 1B**) also remains capable of inhibiting SINV replication (**Figures 3A, B**). Both ZAPS and ZAPL ZnF mutants display reduced antiviral activity, and all other CpG RNA binding cavity mutants display differing degrees of loss of antiviral activity (**Figures 3A, B**). We observed significant differences in viral replication between ZnF mutants and WT for ZAPL (**Figure 3B**) but not for ZAPS (**Figure 3A**), likely due to ZAPL’s greater viral inhibition. In ZAPS, mutants CY and KY have the most significant increase in viral replication compared to ZAPS WT (**Figure 3A**), while ZAPL HFR has the most significant increase in viral replication compared to ZAPL WT (**Figure 3B**).

Meanwhile, we transfected TRIM25 KO 293T cells with TRIM25 RNA binding mutants to assess their ability to inhibit SINV replication. TRIM25 7KA, which binds SINV RNA more robustly than TRIM25 WT (**Figure 1C**) exhibits a similar degree of SINV inhibition (**Figure 3C**). On the other hand, TRIM25 ΔRBD, which fails to bind SINV RNA at all, restores viral replication to TRIM25 KO levels (**Figure 3C**). Finally, we asked whether the observed loss of antiviral activity for any of the ZAP and TRIM25 RNA binding mutants tested was due to a decrease in protein expression. To test this, we assayed protein expression both before and after SINV infection and found that with the exception of the ZAPL ZnF mutants, protein expression for all ZAP and TRIM25 variants increases to varying degrees during infection (**Supplemental Figure 2**). Still, almost all of the ZAP RNA binding mutants express more highly than ZAP-WT (**Supplemental Figures 2A, B**), supporting our hypothesis that it is mutation of these RNA binding residues and not overall protein levels that determines degree of antiviral activity. Together, these data point strongly to the primacy of RNA binding in both ZAP and TRIM25 inhibition of SINV replication.

### ZAP CpG-Mediated RNA Binding but Not TRIM25 RNA Binding Is Required for Inhibition of SINV Translation

**Figure 4:**
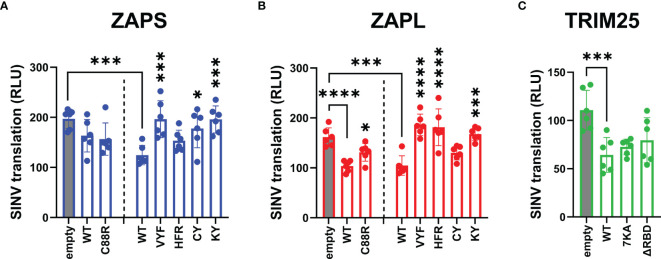
Inhibition of SINV translation by ZAP and TRIM25 RNA binding mutants. **(A, B)** ZAP KO 293T cells or **(C)** TRIM25 KO 293T cells were transfected with **(A)** ZAPS RNA binding mutants, **(B)** ZAPL RNA binding mutants, or **(C)** TRIM25 RNA binding mutants, infected with SINV Toto1101/luc:ts6 at an MOI of 1 PFU/cell, and lysed 6 h.p.i. for measurement of luciferase activity. Data from triplicate wells are combined from two independent experiments. Asterisks indicate statistically significant differences as compared to **(A, B)** ZAP WT, wherein different amounts of **(A)** ZAPS and **(B)** ZAPL WT were transfected to match protein expression levels for each subset of ZnF mutants and CpG RNA binding cavity mutants, or **(C)** TRIM25 WT within each subset of RNA binding mutants (by one-way ANOVA and Dunnett’s multiple comparisons test: *p < 0.05; ***p < 0.001; ****p < 0.0001). Unlabeled comparisons are not significant.

Following our characterization of ZAP and TRIM25 RNA binding mutants’ ability to inhibit SINV replication, we asked whether this antiviral activity stems from a block in alphavirus translation, given that ZAP blocks SINV translation and that TRIM25 is absolutely required for this inhibition (Bick et al., 2003; Li et al., 2017). These prior studies readily measure SINV translation with a replication-deficient temperature-sensitive luciferase reporter virus, Toto1101/luc:ts6, such that any luciferase activity would reflect translation of the incoming viral genome (Rice et al., 1987). Here, we decided to omit the ZnF 4 mutant (H191R) due to its similarity in behavior to the ZnF 2 mutant (C88R), and the CpG RNA binding mutant EKR due to its lack of antiviral activity (**Figures 3A, B**). We transfected ZAP KO 293T cells with ZAPS or ZAPL ZnF 2 and CpG RNA binding cavity mutants and infected with Toto1101/luc:ts6. Here, we found that while transfection of mutants VYF and KY significantly restores SINV translation in both ZAPS and ZAPL (**Figures 4A, B**), HFR only significantly restores SINV translation in the context of ZAPL (**Figure 4B**), in line with its antiviral activity against SINV replication (**Figure 3B**). Given that the residue Y108 is mutated in both VYF and KY and has previously been implicated in determining ZAP CpG specificity (Meagher et al., 2019), our data suggest that Y108 is very important for ZAP inhibition of SINV translation. Moreover, given that HFR largely retains RNA binding in both ZAPS and ZAPL (**Figure 1B**), these data point suggest that mechanisms in addition to RNA binding may modulate ZAP translation inhibition. No dramatic restoration of viral translation is seen for the ZnF 2 mutant (C88R) in either isoform (**Figures 4A, B**), nor does any TRIM25 RNA binding mutant impact TRIM25 inhibition of viral translation when transfected into TRIM25 KO cells (**Figure 4C**), though the lack of effect could be attributed to low protein expression of TRIM25 ΔRBD. Interestingly, we observed that there appear to be two distinct translation phenotypes in the presence of the TRIM25 ΔRBD mutant, wherein half of the replicate wells exhibit inhibited translation similar to TRIM25 WT and the other half have partially restored SINV translation (**Figure 4C**).

We also asked here whether the observed loss of inhibition of viral translation for any of the ZAP and TRIM25 RNA binding mutants tested was due to a decrease in protein expression. Similar to the replication competent SINV Toto1101/luc, infection with the replication-deficient SINV Toto1101/luc:ts6 generally results in higher expression of ZAP and TRIM25 variants (**Supplemental Figure 3**), though the phenotype for a 6 hour infection is not as robust as a 24 hour infection. Interestingly, we observed a mild decrease in expression for ZAPS CY, ZAPS VYF and ZAPL ZnF 2 mutant (C88R) (**Supplemental Figures 3A, B**). Still, most ZAP and TRIM25 RNA binding mutants exhibit similar, if not slightly higher protein expression as compared to ZAP and TRIM25 WT. Together, these data suggest that CpG recognition by ZAP is critical for its alphavirus translation inhibition and this is independent of ZAP isoforms.

### ZAP CpG-Mediated RNA Binding but Not TRIM25 RNA Binding Is Required for Inhibition of JEV Translation, While ZAP ZnF Mutations Enhance JEV Translation Inhibition

Because we observed that specific residues involved in ZAP CpG recognition are more important for SINV translation inhibition, rather than RNA binding in general, we next asked if this trend holds true for ZAP translation inhibition of other viruses. JEV is another virus that is translationally inhibited by ZAP (Chiu et al., 2018). To assess if ZAP and TRIM25 RNA binding play a similar role in blocking JEV translation as they do in blocking alphavirus translation, we assessed the ability of our constructs to inhibit a replication-defective JEV replicon expressing *Renilla* luciferase (Rluc) (Li et al., 2016). Because ZAP can also mediate degradation of JEV RNA, we measured luciferase activity 4 hours after transfecting the JEV replicon reporter to capture a time point that is early enough to see ZAP primarily functioning through translation inhibition, but also long enough to see significant luciferase expression from the replicon. Previous work has shown that there is a minimal decrease in JEV replicon RNA at this time (Chiu et al., 2018). We attempted to co-transfect the JEV replicon with Fluc RNA as a transfection control, but found that ZAPL also restricts Fluc expression (**Supplemental Figure 4**), as previously demonstrated (Li et al., 2019).

**Figure 5:**
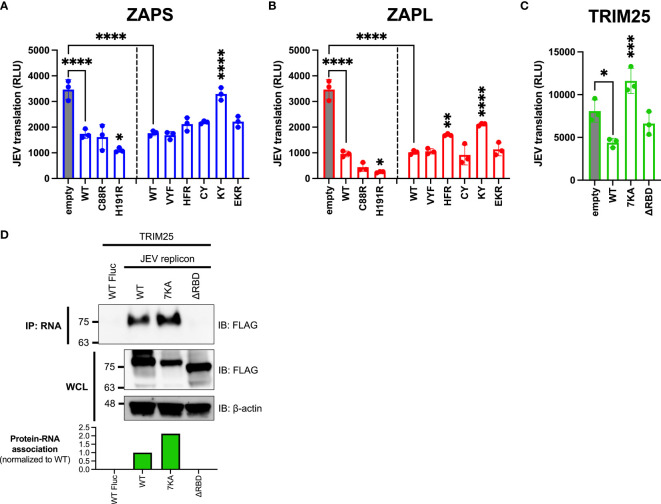
Inhibition of JEV translation by ZAP and TRIM25 RNA binding mutants. **(A, B)** ZAP KO 293T cells or **(C)** TRIM25 KO 293T cells were transfected with **(A)** ZAPS RNA binding mutants, **(B)** ZAPL RNA binding mutants, or **(C)** TRIM25 RNA binding mutants, transfected with a replication-defective Japanese encephalitis virus (JEV) replicon RNA reporter, and lysed 4 hours post-reporter transfection for measurement of luciferase activity. Data from triplicate wells are representative of two independent experiments. Asterisks indicate statistically significant differences as compared to **(A, B)** ZAP WT or **(C)** TRIM25 WT within each subset of RNA binding mutants (by one-way ANOVA and Dunnett’s multiple comparisons test: *p < 0.05; **p < 0.01; ***p < 0.001; ****p < 0.0001). Unlabeled comparisons are not significant. **(D)** TRIM25 KO 293T cells were transfected with TRIM25 mutants. TRIM25 pulled down with RNA and in WCL were assayed by western blot. Blots were quantified with ImageJ. Data are representative of two independent experiments.

We found that the ZAP and TRIM25 RNA binding mutants inhibit JEV translation to varying degrees (**Figure 5**). Surprisingly, we observed that the ZAPS and ZAPL ZnF 4 mutants (H191R) are significantly more inhibitory than their WT counterparts, while the ZAPL ZnF 2 mutant (C88R) is more inhibitory than ZAPL WT (**Figures 5A, B**). Given that these mutants show increased association with TRIM25 (**Figure 2B**), these data suggest a positive relationship between ZAP-TRIM25 interaction and JEV translation inhibition. The ZAP CpG RNA binding cavity mutants either show similar inhibition to ZAPS and ZAPL WT or, in the case of the ZAPS KY and ZAPL HFR and KY mutants, decreased inhibition (**Figures 5A, B**). Taken together with the SINV translation inhibition data, these data further point to the importance of the RNA binding cavities in ZAP inhibition of viral translation, including CpG-specific binding by the residue Y108.

Upon assaying TRIM25 variants, we found that TRIM25 ΔRBD inhibits JEV translation similarly to TRIM25 WT (**Figure 5C**), as we observed with SINV translation inhibition (**Figure 4C**). Surprisingly, the TRIM25 7KA mutant loses its ability to inhibit JEV translation (**Figure 5C**), in contrast to its ability to inhibit SINV translation similarly to TRIM25 WT (**Figure 4C**). Given the diverging phenotypes of the 7KA mutant, we were curious if the TRIM25 mutants show a different pattern of binding to SINV versus JEV replicon RNA that would explain the differences in translation inhibition. We found that the TRIM25 7KA mutant binds JEV replicon RNA to a greater degree than TRIM25 WT, while the TRIM25 ΔRBD mutant loses the ability to bind JEV replicon RNA (**Figure 5D**). These findings recapitulate the TRIM25 SINV RNA binding phenotypes (**Figure 1C**) and suggest that RNA binding by TRIM25 is not an important determinant for its ability to inhibit JEV translation.

### ZAP SINV RNA Binding Negatively Correlates With ZAP-TRIM25 Interaction and Positively Correlates With SINV Replication Inhibition, While ZAP-TRIM25 Interaction Positively Correlates With JEV Translation Inhibition

**Figure 6:**
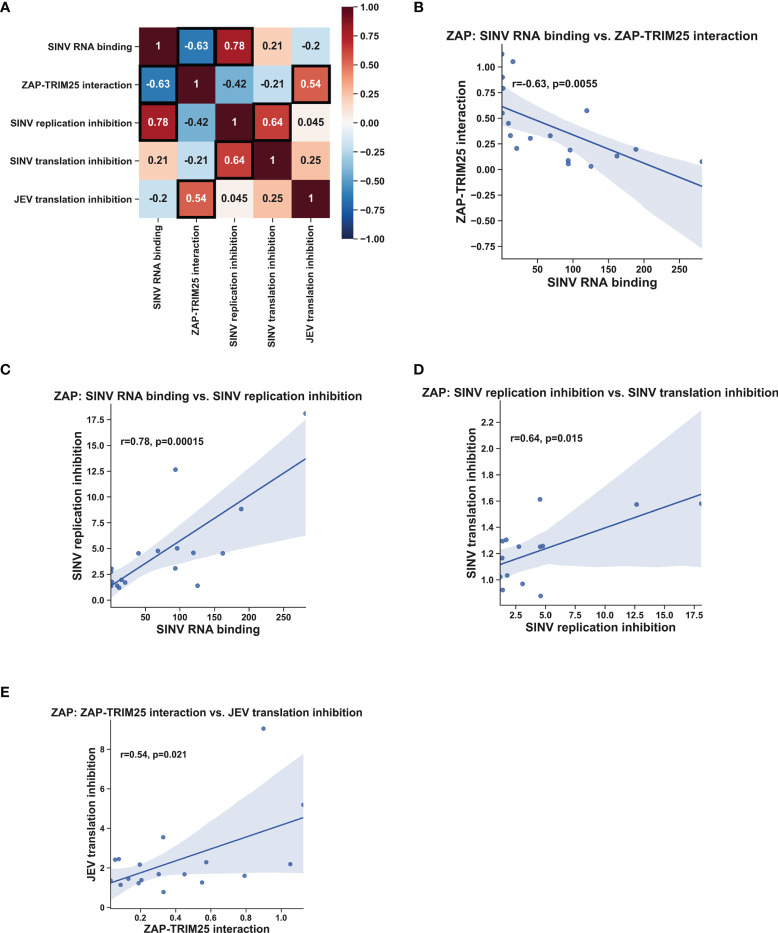
Correlation analysis of ZAP RNA binding mutant phenotypes. Pearson correlation coefficients (r) and p-values (p) were calculated using the SciPy package in Python and plotted using the Matplotlib and Seaborn packages. Pearson correlation coefficients are summarized in a heat map **(A)**. Boxed coefficients have statistically significant p-values (p < 0.05). Pairwise correlations with significant p-values from **(A)** are shown in **(B–E)**.

Given our wide panel of ZAPS and ZAPL RNA binding mutants, we sought to look for significant correlations between any pairs of ZAP phenotypes. To facilitate this, we quantified the immunoblots for ZAP SINV RNA binding and ZAP-TRIM25 co-IPs and calculated fold inhibition relative to empty plasmid transfection for the viral inhibition assays (**Table 1**). Using these values, we calculated Pearson correlation coefficients and p-values for each pairwise phenotype comparison. When analyzing our data for the ZnF 2 and 4 mutants and the complete panel of CpG RNA binding cavity mutants (**Figure 6A**), we found a significant negative correlation (r = -0.63, p<0.01) between ZAP SINV RNA binding and ZAP-TRIM25 interaction (**Figure 6B**), lending further support to the idea that ZAP RNA binding competes with its ability to associate with TRIM25. We also observed a significant positive correlation (r = 0.78, p<0.001) between ZAP SINV RNA binding and SINV replication inhibition (**Figure 6C**), but interestingly, no correlation between ZAP SINV RNA binding and SINV translation inhibition (**Table 1**). This suggests that while general ZAP RNA binding is important for its antiviral activity, it is not as critical for the specific step of translation inhibition. Finally, we found a significant positive correlation (r = 0.64, p<0.05) between ZAP-TRIM25 interaction and JEV translation inhibition (**Figure 6D**), further suggesting that ZAP interaction with TRIM25 potentiates its ability to inhibit JEV translation. Because we had a limited number of mutants for TRIM25, we were not able to find significant correlations between any pair of TRIM25 phenotypes (data not shown).

**Table 1:** Quantified values for ZAP RNA binding mutant phenotypes.

Isoform	Mutant	SINV RNA binding	ZAP-TRIM25 interaction	SINV replication inhibition	SINV translation inhibition	JEV translation inhibition
ZAPS	WT for ZnF mutants	161.914	0.131	4.545	1.252	1.446
ZAPS	C88R	9.083	0.450	1.395	1.295	1.676
ZAPS	H191R	15.452	1.052	1.951	N/A	2.184
ZAPS	WT for cavity mutants	40.011	0.304	4.542	1.613	1.684
ZAPS	V72A/Y108A/F144A	1.444	0.792	1.777	1.033	1.598
ZAPS	H176A/Y184A/R189A	20.797	0.205	1.706	1.305	1.381
ZAPS	C96A/Y98A	0.408	0.550	1.361	1.166	1.260
ZAPS	K107A/Y108A	11.959	0.330	1.190	1.023	0.780
ZAPS	E148A/K151A/R170A	95.909	0.189	5.015	N/A	1.226
ZAPL	WT for ZnF mutants	93.414	0.055	12.652	1.574	2.416
ZAPL	C88R	0.034	1.126	2.756	1.253	5.191
ZAPL	H191R	0.761	0.899	3.043	N/A	9.046
ZAPL	WT for cavity mutants	281.812	0.076	18.090	1.580	2.444
ZAPL	V72A/Y108A/F144A	119.441	0.574	4.590	0.878	2.281
ZAPL	H176A/F184A/R189A	125.392	0.030	1.396	0.922	1.357
ZAPL	C96A/Y98A	68.060	0.329	4.774	1.257	3.551
ZAPL	K107A/Y108A	93.185	0.086	3.071	0.969	1.138
ZAPL	E148A/K151A/R170A	188.614	0.196	8.828	N/A	2.165

Immunoblots for ZAP SINV RNA binding assays and ZAP-TRIM25 co-IPs were quantified in ImageJ, with higher values indicating increased RNA or TRIM25 interaction. Fold inhibition values for viral inhibition assays were calculated relative to empty plasmid transfection, with higher values indicating greater viral replication or translation inhibition. N/A: SINV translation inhibition data on ZAP H191R and EKR mutants were not collected for analysis. Data are combined from two independent experiments.

## Discussion

Though the RNA binding functions of ZAP and TRIM25 have been previously dissected, in this study we placed these functions in a wider context of viral translation inhibition. We found that ZAP and TRIM25 RNA binding domains contribute in varying degrees to their ability to bind SINV genomic RNA. We also observed that reduction of RNA binding by ZAP and TRIM25 generally increases their ability to interact with each other while reducing both of their abilities to inhibit SINV replication. When looking at viral translation inhibition more specifically, we found that the function of the ZAP CpG RNA binding cavities is most important for SINV and JEV translation inhibition, while general ZAP RNA binding and TRIM25 RNA binding is less critical. Some of our findings diverge from those observed previously, demonstrating the importance of studying ZAP and TRIM25 functions in a more biologically relevant context with the presence of full-length viral RNA.

Our SINV genomic RNA binding assays mostly recapitulate the results of prior studies, which investigated binding to synthetic viral RNA fragments or RNA constructs. Notably, we showed that all four ZnFs are individually required for efficient ZAP binding to SINV RNA (**Figure 1A**), while previously, only ZnF 2 has been demonstrated to be required for binding to SINV RNA fragments (Guo et al., 2004; Wang et al., 2010). Our findings also diverge from previous studies for the ZAP EKR CpG binding cavity mutant, which has reduced binding to a 6-nt single-stranded RNA probe (Luo et al., 2020); we showed that the ZAPS and ZAPL EKR mutants bind SINV RNA to a greater degree than their WT counterparts (**Figure 1B**). These contrasting findings may be a result of the different assays used in our studies and suggest that the function of the CpG-specific binding residues within ZnF 3 varies based on its specific RNA target. We also found that impairing ZAP RNA binding results in increased association with TRIM25 (**Figures 2A, B**, **Figure 6B**), consistent with previous findings (Gonçalves-Carneiro et al., 2021). Given that ZAP has also been shown to interact with TRIM25 through its N-terminal ZnFs, we hypothesize that RNA binding may compete with ZAP-TRIM25 interaction, though further experiments are required to test this hypothesis.

As expected, we found that the ZAP RNA binding mutants show deficiencies in their ability to inhibit SINV viral replication and translation (**Figures 3**, **4**) recapitulating previous findings on their inhibition of SINV viral production and luciferase expression from reporters containing ZAP-sensitive fragments (Guo et al., 2004; Chen et al., 2012; Luo et al., 2020). When looking at SINV translation inhibition specifically, certain residues appear to be more critical for inhibition, particularly the CpG RNA binding mutants with the mutation Y108A (**Figures 4A, B**). Taken with our finding of a significant positive correlation between ZAP SINV RNA binding and SINV replication inhibition (**Figure 6B**), but no correlation between SINV RNA binding and SINV translation inhibition (**Table 1**), our data suggests that general ZAP RNA binding is less important than binding by particular residues for the specific step of translation inhibition. We observed a similar rescue of JEV translation inhibition with certain CpG RNA binding cavity mutants (**Figures 5A, B**), pointing to the importance of the ZAP CpG RNA binding cavities in facilitating translation inhibition of diverse viruses.

Deletion of all four ZnFs eliminates ZAP’s ability to restrict JEV (Chiu et al., 2018), but our data suggests that mutation of ZnF 2 (C88R) and 4 (H191R) individually increases antiviral activity (**Figures 5A, B**), potentially due to these mutants’ increased ability to interact with TRIM25 (**Figures 2A, B**). Consistent with this, we found a significant positive correlation between ZAP’s ability to interact with TRIM25 and its inhibition of JEV translation (**Figure 6D**). We speculate that ZAP-TRIM25 interaction might potentiate TRIM25 recruitment and ubiquitination of cellular substrates important for mediating JEV translation inhibition and/or RNA degradation, since ZAP can also mediate degradation of JEV RNA (Chiu et al., 2018). One possible such substrate is KHNYN, which functions in viral RNA degradation by ZAP and requires TRIM25 for its antiviral activity (Ficarelli et al., 2019). Additional studies are needed to evaluate these hypotheses.

By studying ZAP RNA binding mutations in both full-length ZAPS and ZAPL, we uncovered isoform differences in their effects on viral inhibition. The HFR CpG RNA binding cavity mutant shows an impaired ability to inhibit replication of SINV and translation of SINV and JEV in ZAPL, but not in ZAPS (**Figures 3**–**5**). ZAPL contains a catalytically inactive C-terminal PARP-like domain (Kerns et al., 2008) and a prenylation motif within this domain that targets it to endosomal membranes (Schwerk et al., 2019; Kmiec et al., 2021), which together may alter the ability of ZAPL to bind RNA, interact with other cellular or viral proteins, and inhibit viral translation in different viral contexts. Additional mutagenesis studies targeting the PARP-like domain are needed to tease out its role in mediating RNA binding. Because we introduced our mutants into ZAP KO cells, further work is also needed to address the possibility that the function of ZAP RNA binding mutants could be modulated by interactions between ZAPS and ZAPL, as well as with the additional isoforms ZAPM and ZAPXL (Li et al., 2019).

For TRIM25, we found that the ΔRBD mutant has lost the ability to bind SINV and JEV replicon RNA (**Figures 1C**, **5D**). On the other hand, the TRIM25 7KA mutant shows increased binding to SINV and JEV replicon RNA, as well as the Fluc control RNA that is not bound by TRIM25 WT (**Figures 1C**, **5D**). Previously, the TRIM25 7KA mutant was shown to be deficient in binding to a short double-stranded RNA probe (Sanchez et al., 2018). Similar to the ZAPS EKR mutant, we speculate that our divergent findings on the TRIM25 7KA mutant may result from differences in assays, and that the binding function of the 7K motif may depend on the specific RNA target. In our hands, the TRIM25 ΔRBD mutant is less stable than the other TRIM25 constructs, and so its expression is likely too low to be detected in the ZAP co-IP (**Figure 2C**). We did observe that the TRIM25 7KA mutant shows a more robust interaction with ZAPS and ZAPL than TRIM25 WT (**Figure 2C**). Taken together with the increased binding of this mutant to SINV RNA, our results suggest that RNA and ZAP binding are not inversely related for TRIM25, unlike for ZAP. While the TRIM25 ΔRBD mutant has lost its ability to restrict SINV replication (**Figure 3C**), it is still able to inhibit SINV translation similarly to TRIM25 WT despite some variability across replicates (**Figure 4C**). We hypothesize that these divergent phenotypes for SINV replication and translation result from the variations in the TRIM25 ΔRBD mutant expression, rather than a defect in activity. While the 7K motif has been shown to be required for TRIM25 inhibition of dengue virus (Sanchez et al., 2018), which is not sensitive to ZAP inhibition (Chiu et al., 2018), our observations suggest that it is dispensable in the context of ZAP-mediated viral translation inhibition. In fact, given that the TRIM25 7KA mutant loses the ability to inhibit JEV translation (**Figure 5C**) and binds JEV replicon RNA more robustly than TRIM25 WT (**Figure 5D**), excess binding of TRIM25 to its target RNA may even impede viral translation inhibition in certain contexts. It is possible that an increased presence of TRIM25 may hinder RNA or TRIM25 interactions with co-factors that function in JEV translation inhibition, but not SINV translation inhibition; further work is required to test this hypothesis. Taken together, our results indicate that while RNA binding is important for TRIM25’s general ability to inhibit SINV replication, it is likely not required for translation inhibition specifically.

Overall, our findings suggest while ZAP RNA binding is required for its antiviral activity, its ability to specifically recognize CpG dinucleotides in viral RNA is more critical in the process of viral translation inhibition. Additionally, ZAP RNA binding and interaction with TRIM25 may represent two distinct determinants for ZAP antiviral activity in varying viral contexts. Altogether, our study has shed more light on the roles of viral RNA binding and co-factor dependency in the mechanism of ZAP translation inhibition and raised interesting questions on the requirement of specific residues for ZAP and TRIM25 RNA binding, protein-protein interaction, and antiviral activity.

## Materials and Methods

### Cell Culture, Viruses, and Infections

Dr. Akinori Takaoka at Hokkaido University generously provided ZAP KO 293T cells (clone 89) and its parental 293T lines (Hayakawa et al., 2011). TRIM25 KO 293T cells were generated using CRISPR-Cas9 as previously described (Li et al., 2017; unpublished data). Cells were cultured in Dulbecco’s Modified Eagle Medium (DMEM, Thermo Fisher Scientific) with 10% fetal bovine serum (FBS) added.

Infections with SINV expressing firefly luciferase (Toto1101/Luc) and temperature-sensitive SINV (Toto1101/Luc:ts6) have been previously described (Rice et al., 1987; Bick et al., 2003). Each independent experiment included triplicate wells of biological replicates per condition. BHK-21 cells were used to generate viral stocks and titers for multiplicity of infection calculations (Bick et al., 2003).

### Plasmids and Transfections

The replication-defective JEV replicon plasmid was generously provided by Dr. Bo Zhang at the Wuhan Institute of Virology (Li et al., 2016). The plasmid pcDNA3.1-3XFLAG was kindly gifted to us by Dr. Oliver Fregoso at UCLA. To generate pcDNA3.1-myc, a myc tag was swapped in for the 3XFLAG tag using *BamHI* and *HindIII* restriction sites. ZAPS and ZAPL were cloned into pcDNA3.1-3XFLAG from pTRIP-TagRFP-hZAPS and pTRIP-TagRFP-hZAPL (Li et al., 2017), respectively, using *NotI* and *XbaI* restriction sites. Dr. Jae U. Jung at the University of Southern California generously provided full-length TRIM25 (Gack et al., 2007). TRIM25 was cloned into both pcDNA3.1-3XFLAG and pcDNA3.1-myc using *XhoI* and *XbaI* restriction sites. All ZAP and TRIM25 constructs are myc- or 3XFLAG-tagged on the N-terminal end.

Point mutations in ZAPS and ZAPL were generated using the Q5 Site-Directed Mutagenesis Kit (New England Biolabs) and all plasmids were verified by sequencing (Genewiz). Primers for mutations were synthesized by Integrated DNA Technologies (**Supplementary Table 1**). The TRIM25 ΔRBD mutant (Choudhury et al., 2017) was generated by overlapping PCR (**Supplementary Table 1**). The TRIM25 7KA mutant was generated by ordering a gBlocks Gene Fragment from IDT with all lysines in _381_KKVSKEEKKSKK_392_ mutated to alanines (Sanchez et al., 2018), and utilizing innate restriction sites in TRIM25 “*BsrGI* and *BamHI* (underlined)”, to replace wild-type sequence in TRIM25. The 7KA mutated sequence is bolded in the below gene block, and nonessential nucleotides on the 5’ and 3’ ends are written in lowercase.

5’gtttTGTACAGTCAGATCAACGGGGCGTCGAGAGCACTGGATGATGTGAGAAACAGGCAGCAGGATGTGCGGATGACTGCAAACAGAAAGGTGGAGCAGCTACAACAAGAATACACGGAAATGAAGGCTCTCTTGGACGCCTCAGAGACCACCTCGACAAGGAAGATAAAGGAAGAGGAGAAGAGGGTCAACAGCAAGTTTGACACCATTTATCAGATTCTCCTCAAGAAGAAGAGTGAGATCCAGACCTTGAAGGAGGAGATTGAACAGAGCCTGACCAAGAGGGATGAGTTCGAGTTTCTGGAGAAAGCATCAAAACTGCGAGGAATCTCAACAAAGCCAGTCTACATCCCCGAGGTGGAACTGAACCACAAGCTGATAAAAGGCATCCACCAGAGCACCATAGACCTCAAAAACGAGCTGAAGCAGTGCATCGGGCGGCTCCAGGAGCCCACCCCCAGTTCAGGTGACCCTGGAGAGCATGACCCAGCGTCCACACACAAATCCACACGCCCTGTG**GCAGCAGTCTCCGCAGAGGAAGCAGCATCCGCAGCA**CCTCCCCCTGTCCCTGCCTTACCCAGCAAGCTTCCCACGTTTGGAGCCCCGGAACAGTTAGTGGATTTAAAACAAGCTGGCTTGGAGGCTGCAGCCAAAGCCACCAGCTCACATCCGAACTCAACATCTCTCAAGGCCAAGGTGCTGGAGACCTTCCTGGCCAAGTCCAGACCTGAGCTCCTGGAGTATTACATTAAAGTCATCCTGGACTACAACACCGCCCACAACAAAGTGGCTCTGTCAGAGTGCTATACAGTAGCTTCTGTGGCTGAGATGCCTCAGAACTACCGGCCGCATCCCCAGAGGTTCACATACTGCTCTCAGGTGCTGGGCCTGCACTGCTACAAGAAGGGGATCCgttt-3’

X-tremeGENE9 DNA Transfection Reagent (Roche Life Science) was used to transfect cells at a ratio of 3 μL to 1 μg DNA according to the manufacturer’s instructions. To keep the total plasmid amount in co-transfections constant, empty vectors pcDNA3.1-myc or 3XFLAG were transfected as necessary (6 well plate, 2 μg total input; 24 well plate, 250 ng total input).

### *In vitro* Transcription

SINV DNA templates for transcription were generated by *XhoI* linearization of pToto1101 (Rice et al., 1987). SINV RNA was transcribed *in vitro* by Sp6 RNA polymerase (New England Biolabs) in the presence of the cap analog [m7G(5’)ppp(5’)G] (New England Biolabs). Fluc DNA templates for transcription were amplified from the pGL3-Control plasmid (Promega). Fluc RNA was transcribed *in vitro* using the mMESSAGE mMACHINE T7 Transcription Kit (Invitrogen). Biotin-labeled RNAs were generated by adding 10mM biotin-16-UTP (Roche Life Science) to *in vitro* transcription reactions. JEV replicon DNA templates for transcription were generated by *XhoI* linearization and transcribed *in vitro* using the mMESSAGE mMACHINE T7 Transcription Kit (Invitrogen). Transcribed RNAs were purified using the *Quick-*RNA Miniprep Kit (Zymo Research) and biotinylation was confirmed by streptavidin dot blot (Chan et al., 2020).

### *In vitro* RNA Pull-Down Assay

0.4 pmol of biotin-labeled SINV or Fluc RNA probes were heated for 2 min at 90°C, chilled on ice for 2 min, and incubated with 50 μL 2x RNA structure buffer for 30 min at room temperature to ensure proper secondary RNA structure formation, as previously described (Bai et al., 2016). *In vitro* RNA pull-down was then performed as previously described (Chiu et al., 2018). In brief, RNA probes were incubated with 100 μg of lysates from ZAP or TRIM25 KO 293T cells transfected with ZAP or TRIM25 constructs for 48 hours. Cell extracts were lysed by CHAPS lysis buffer [10 mM Tris-HCl (pH 7.4), 1 mM MgCl_2_, 1 mM EGTA, 0.5% CHAPS, 10% glycerol, and 5 mM 2-mercaptoethanol] with complete protease inhibitor cocktail (Roche Life Science) and incubated with RNA probes in a final volume of 100 μL RNA binding buffer supplemented with 1 unit/μL RNAseOUT (Thermo Fisher Scientific), 1 μg/μL heparin (Sigma-Aldrich), and 100 ng/μL yeast tRNA (ThermoFisher) for 30 min at 30°C. Lysate-RNA mixtures were then incubated with 300 μL of Dynabeads M-280 Streptavidin (Invitrogen) for 30 min at room temperature on a shaker. Protein-RNA complexes were washed three times by RNA binding buffer, and proteins were eluted by incubation with 30 μL of 4x Laemmli Sample Buffer (Bio-Rad) for 5 min at 95°C. The proteins were further analyzed by immunoblot and quantified by ImageJ as previously described (Davarinejad, 2015). Briefly, to calculate the quantity of ZAP or TRIM25 bound to RNA relative to input ZAP or TRIM25 protein, the net value of the FLAG (ZAP or TRIM25) band in the whole cell lysate was divided by the net value of the β-actin loading control band in the whole cell lysate, giving a normalized input value. The net value of the FLAG band in the RNA IP band was then divided by the normalized input value.

### Immunoblot Analysis

Proteins were resolved through SDS-PAGE using NuPAGE MOPS SDS running buffer (Thermo Fisher Scientific) and 4-15% precast Mini-PROTEAN TGX Gels (Bio-Rad) before transferring to a PVDF membrane (Bio-Rad). Immunodetection was achieved with 1:2,500 anti-myc (Cell Signaling Technology), 1:20,000 anti-FLAG (Sigma-Aldrich), and 1:20,000 anti-actin-HRP (Sigma-Aldrich). Primary antibodies were detected with 1:20,000 goat anti-mouse HRP (Jackson ImmunoResearch) or 1:20,000 goat anti-rabbit HRP (Thermo Fisher Scientific). Proteins were visualized on a ChemiDoc (Bio-Rad) using ProSignal Pico ECL Reagent (Genesee Scientific). ImageJ was used to quantify western blots as previously described (Davarinejad, 2015). Briefly, protein band intensities for each blot were measured by taking the net grey mean value of each band. The net grey mean value is defined as the inverted pixel density (255 – grey mean value) of a band with the inverted pixel density of the background (defined as an equivalent area of the blot above or below the band) subtracted.

### Co-Immunoprecipitation Assay

To assess ZAP or TRIM25 co-immunoprecipitation (co-IP) with RNA binding mutants of TRIM25 or ZAP, respectively, cells were transfected in 6-well plates, collected, and then lysed by rotating in FLAG IP buffer (100 mM Tris-HCl 8.0, 150 mM NaCl, 5 mM EDTA, 1 mM DTT, 5% glycerol, 0.1% NP-40) supplemented with a complete protease inhibitor cocktail (Roche Life Science) at 4°C for 30 min, before spinning down at 14,000 rpm at 4°C for 15 min. To equilibrate beads prior to use, anti-FLAG beads (EZview™ Red ANTI-FLAG M2 Affinity Gel, Sigma-Aldrich) or anti-myc beads (EZview™ Red Anti-c-Myc Affinity Gel, Sigma-Aldrich) were washed 3 times in FLAG IP buffer. Three hundred μL of whole cell lysate (WCL) were incubated with 30 μL of anti-FLAG or -myc beads rotating at 4˚C for 45 minutes. FLAG IP buffer was used to wash immunoprecipitates 3 times before eluting bound proteins with SDS loading buffer, and boiling for 5 minutes for immunoblot analysis. Western blot ImageJ analysis was performed as previously described (Davarinejad, 2015). Briefly, to calculate the relative quantity of ZAP RNA binding mutants in the myc (TRIM25) co-IP, the net value of the FLAG (ZAP) IP band, defined as the net grey mean value of ZAP alone subtracted from each mutant band, was divided by the net value of the myc IP for each mutant. To calculate the relative quantity of TRIM25 RNA binding mutants in the FLAG (ZAP) co-IP, the net value of the myc (TRIM25) IP band, defined as the net grey mean value of TRIM25 alone subtracted from each mutant band, was divided by the net value of the FLAG IP for each mutant. To account for the different expression levels of TRIM25 mutants, the myc IP band was first divided by the net grey mean value of the myc WCL band for each mutant, normalized to the value of the band for TRIM25 alone.

### JEV Replicon Reporter Assay

Following transfection of ZAP or TRIM25 constructs into ZAP or TRIM25 KO 293T for 48 hours, JEV replicon RNA was transfected by *Trans*IT-mRNA Transfection Kit (Mirus Bio). Cells were lysed 4 hours post-transfection of replicon RNA and luciferase activity was measured by Dual-Luciferase Reporter Assay (Promega). Each independent experiment included triplicate wells of biological replicates per condition.

### Statistical Analysis

Statistical analyses in **Figures 3**–**5** were performed on biological replicates from triplicate wells using GraphPad Prism. Statistical analyses in **Figure 6** were performed using the SciPy package in Python and visualized using the Matplotlib and Seaborn packages (Hunter, 2007; Virtanen et al., 2020; Waskom, 2021).

## Data Availability Statement

The original contributions presented in the study are included in the article/**Supplementary Material**. Further inquiries can be directed to the corresponding author.

## Author Contributions

EY, LN, and ML conceptualized and designed the study. CW and RK assisted in cloning ZAP and TRIM25 RNA binding mutants. LN performed RNA binding and JEV replicon experiments, while EY performed co-IP and SINV replication and translation experiments. LN performed the correlation analysis. EY and LN co-wrote the first draft of the manuscript. ML provided critical feedback. All authors contributed to manuscript revision, read, and approved the submitted version.

## Funding

This work was supported in part by NIH R01AI158704 (ML), UCLA AIDS Institute and Charity Treks 2019 Seed Grant (ML), Ruth L. Kirschstein Multidisciplinary Training Grant in Microbial Pathogenesis (NRSA AI007323; EY), Ruth L. Kirschstein Cellular and Molecular Biology Training Program (NRSA GM007185; LN), Warsaw Fellowship (EY), and Whitcome Fellowship (EY, LN).

## Conflict of Interest

The authors declare that the research was conducted in the absence of any commercial or financial relationships that could be construed as a potential conflict of interest.

## Publisher’s Note

All claims expressed in this article are solely those of the authors and do not necessarily represent those of their affiliated organizations, or those of the publisher, the editors and the reviewers. Any product that may be evaluated in this article, or claim that may be made by its manufacturer, is not guaranteed or endorsed by the publisher.

## Supplementary Material

Supplementary Figure 1 Expression of ZAP and TRIM25 mutants. **(A-E)** Final transfected DNA amounts for each mutant written below, where 1k = 1 μg of DNA. **(A)** Western blot of ZAPS zinc finger (ZnF) mutants. **(B)** Western blot of ZAPL ZnF mutants. **(C)** Western blot of ZAPS CpG RNA binding cavity mutants. **(D)** Western blot of ZAPL CpG RNA binding cavity mutants. Yellow highlighted cells indicate the selected amount to transfect for subsequent experiments. **(E)** Western blot of TRIM25 RNA binding mutants.

Supplementary Figure 2 Effect of SINV infection on ZAP and TRIM25 protein expression. **(A, B)** ZAP KO 293T cells or **(C)** TRIM25 KO 293T cells were transfected with **(A)** ZAPS RNA binding mutants, **(B)** ZAPL RNA binding mutants, or **(C)** TRIM25 RNA binding mutants. Lysates were harvested at 0 and 24 h.p.i. with SINV Toto1101/luc infection at an MOI of 0.01 PFU/cell. Data from triplicate wells are representative of two independent experiments

Supplementary Figure 3 Effect of replication-deficient SINV infection on ZAP and TRIM25 protein expression. **(A, B)** ZAP KO 293T cells or **(C)** TRIM25 KO 293T cells were transfected with **(A)** ZAPS RNA binding mutants, **(B)** ZAPL RNA binding mutants, or **(C)** TRIM25 RNA binding mutants. Lysates were harvested at 0 and 6 h.p.i. with SINV Toto1101/luc:ts6 infection at an MOI of 1 PFU/cell. Data from triplicate wells are representative of two independent experiments.

Supplementary Figure 4 Sensitivity of the firefly luciferase control RNA to ZAP. **(A, B)** ZAP KO 293T cells or **(C)** TRIM25 KO 293T cells were transfected with **(A)** ZAPS RNA binding mutants, **(B)** ZAPL RNA binding mutants, or **(C)** TRIM25 RNA binding mutants, transfected with firefly luciferase (Fluc) RNA, and lysed 4 hours post-RNA transfection for measurement of luciferase activity. Data from triplicate wells are representative of two independent experiments. Asterisks indicate statistically significant differences as compared to **(A, B)** ZAP WT or **(C)** TRIM25 WT within each subset of RNA binding mutants (by one-way ANOVA and Dunnett’s multiple comparisons test: *p<0.05; **p<0.01; ***p<0.001). Unlabeled comparisons are not significant.
