# Supplementary figures and images for: The Role of ZAP and TRIM25 RNA Binding in Restricting Viral Translation
Source: Front Cell Infect Microbiol. 2022 Jun 21;12:886929. doi: 10.3389/fcimb.2022.886929 (PMC9253567; doi:10.3389/fcimb.2022.886929)

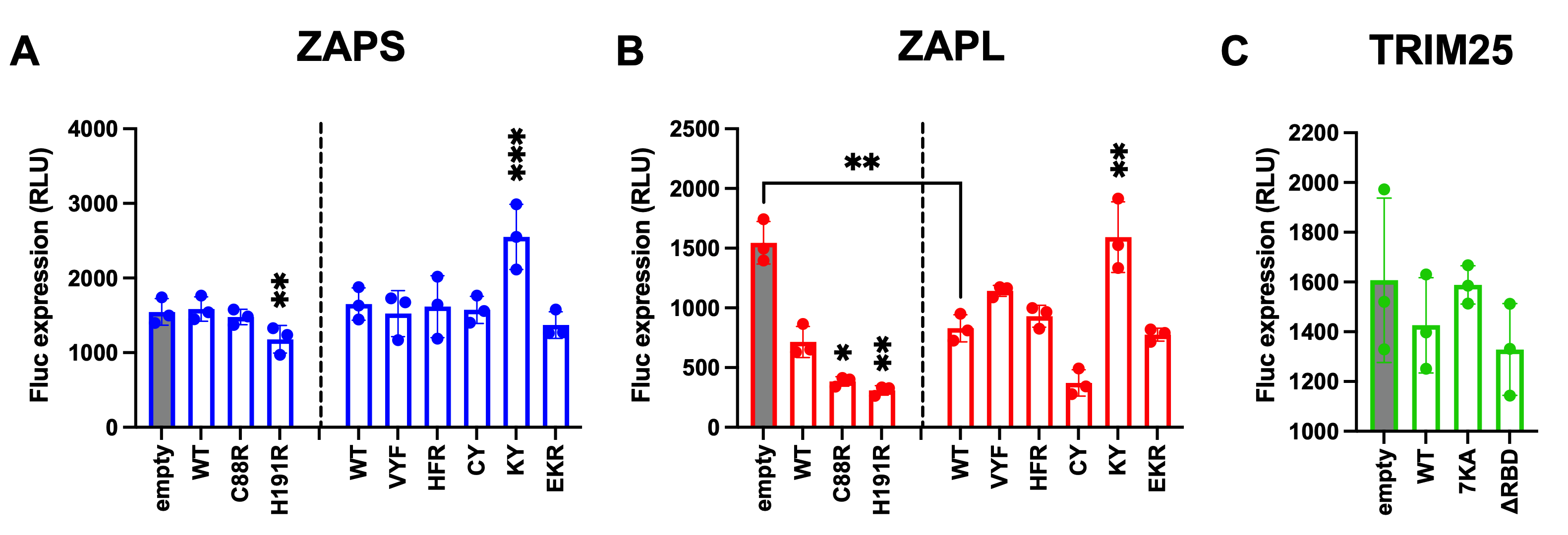

Supplement: Supplementary Figure 4 — Sensitivity of the firefly luciferase control RNA to ZAP. (A, B) ZAP KO 293T cells or (C) TRIM25 KO 293T cells were transfected with (A) ZAPS RNA binding mutants, (B) ZAPL RNA binding mutants, or (C) TRIM25 RNA binding mutants, transfected with firefly luciferase (Fluc) RNA, and lysed 4 hours post-RNA transfection for measurement of luciferase activity. Data from triplicate wells are representative of two independent experiments. Asterisks indicate statistically significant differences as compared to (A, B) ZAP WT or (C) TRIM25 WT within each subset of RNA binding mutants (by one-way ANOVA and Dunnett’s multiple comparisons test: *p<0.05; **p<0.01; ***p<0.001). Unlabeled comparisons are not significant. [file Image_4.tiff]
