# Supplementary material for: The Role of ZAP and TRIM25 RNA Binding in Restricting Viral Translation
Source: Front Cell Infect Microbiol. 2022 Jun 21;12:886929. doi: 10.3389/fcimb.2022.886929 (PMC9253567; doi:10.3389/fcimb.2022.886929)
Supplement: Supplementary file 5 [file Table_1.docx]

**Supplementary Table 1. Primers used to generate ZAP and TRIM25 RNA binding mutants.** Additional features pertinent to primer design, such as additional mutations, are noted as necessary.

| **RNA binding mutant** | **Mutation** | **Sequence (5’-3’)** | **Forward (F)/Reverse (R)** | **Included features** |
| --- | --- | --- | --- | --- |
| ZAP ZnF1 | H86K | GAGACCCTGCGATAACCTGAAGCTCTGCAAACTCAACTTGC | F |  |
|  |  | GCAAGTTGAGTTTGCAGAGCTTCAGGTTATCGCAGGGTCTC | R |  |
| ZAP ZnF2 | C88R | GCGATAACCTGCATCTCCGCAAACTCAACTTGCTG | F |  |
|  |  | CAGCAAGTTGAGTTTGCGGAGATGCAGGTTATCGC | R |  |
| ZAP ZnF3 | C168R | ACCAGCAGCCACCGCGTTCAAGACTCCAC | F |  |
|  |  | GTGGAGTCTTGAACGCGGTGGCTGCTGGT | R |  |
| ZAP ZnF4 | H191R | CTGCCTCCGGTCCCGTAACCTGATGGACA | F |  |
|  |  | TGTCCATCAGGTTACGGGACCGGAGGCAG | R |  |
| ZAP VYF | V72A | TTGCGACGGCAGGCCCGGGCTCTAG | F |  |
|  |  | CTAGAGCCCGGGCCTGCCGTCGCAA | R |  |
|  | Y108A | GTCCGAGCGGAATTTATGCAAAGCTTCTCATGAGGTTCTCTCAG | F |  |
|  |  | CTGAGAGAACCTCATGAGAAGCTTTGCATAAATTCCGCTCGGAC | R |  |
|  | F144A | CCTCCTCCAAAGTGATGCTTTTATGCCCGAGATATGC | F |  |
|  |  | GCATATCTCGGGCATAAAAGCAGGATCACTTTGGAGGAGG | R |  |
| ZAP HFR | H176A | CATCTGTGACGCCTTCACCCGAGG | F |  |
|  |  | TGGAGTCTTGAACACGGT | R |  |
|  | F184A | ACCCGAGGGAACTGTCGTGCTCCCAACTGCCTC | F |  |
|  |  | GAGGCAGTTGGGAGCACGACAGTTCCCTCGGGT | R |  |
|  | R189A | CCATCAGGTTATGGGACGCGAGGCAGTTGGGAAAAC | F |  |
|  |  | GTTTTCCCAACTGCCTCGCGTCCCATAACCTGATGG | R |  |
| ZAP CY | C96A | GCTGGGCCGGGCCAACTATTCGC | F |  |
|  |  | AAGTTGAGTTTGCAGAGATGC | R |  |
|  | Y98A | CCGGGCCAACGCATCGCAGTCCG | F | Includes C96A mutation |
|  |  | CCCAGCAAGTTGAGTTTG | R |  |
| ZAP KY | K107A | GAATTTATGCGCATATTCTCATGAGGTTCTCTCAG | F |  |
|  |  | CGCTCGGACTGCGAATAG | R |  |
|  | Y108A | TTTATGCGCAGCCTCTCATGAGGTTCTCTCAGAAGAG | F | Includes K107A mutation |
|  |  | TTCCGCTCGGACTGCGAA | R |  |
| ZAP EKR | E148A | TTTATGCCCGCGATATGCAAAAG | F |  |
|  |  | AAAAGGATCACTTTGGAG | R |  |
|  | K151A | CGCGATATGCGCAAGTTATAAGG | F | Includes E148A mutation |
|  |  | GGCATAAAAAAAGGATCAC | R |  |
|  | R170A | ACCGTGTTCAGCACTCCACATCTG | F |  |
|  |  | GGCTGCTGGTTACAAATC | R |  |
| TRIM25ΔRBD | TRIM25 aa 1-469 | gttt*CTCGAGGTATCG*ATATGGCAGAGCTGTGCCCC | F | Includes XhoI and ClaI sites (italicized); nonessential sequence in lowercase |
|  |  | TTTGTTGTGGGCGGTGTTGTAGTC | R | Includes sequence upstream of the deletion (underlined, nt 1384-1407) |
|  | TRIM25 aa 464-469, 509-630 | CAACACCGCCCACAACAAA**AAGGGGATCCACTACTGGGA** | F | Includes TRIM25 sequence upstream (underlined, nt 1384-1407) and downstream of the deletion (bolded, nt 1525-1544) |
|  |  | gttt*TCTAGAGCGGCCGCC*TA | R | Includes XbaI and NotI sites (italicized); nonessential sequence in lowercase |
